# ADVANCING HEALTH EQUITY AND REDUCING DISPARITIES: COMMUNITY-IDENTIFIED AND DRIVEN PROJECTS

**DOI:** 10.1093/geroni/igae098.0729

**Published:** 2024-12-31

**Authors:** Robbin Frazier, Elma Johnson

**Affiliations:** University of Minnesota, Minneapolis, Minnesota, United States; University of Minnesota, Minneapolis, Minnesota, United States

## Abstract

Soon after its re-launch in 2021, the Center for Healthy Aging and Innovation (CHAI) at the University of Minnesota established its Equity and Community Engagement (ECE) Core to develop the infrastructure for community engagement in CHAI and build relationships with a variety of community partners serving older adults in BIPOC and underserved communities. After careful planning and a thoughtful recruitment process, the ECE Core launched CHAI’s first Community Advisory Board (CAB) in the fall of the same year. This diverse group brings together older adults from the largest ethnic groups in the state, and provides input, wisdom and lived experience to guide CHAI’s work and priorities. Two years after its launch, the CAB has identified key community needs related to aging and started work on several community-led projects to address ageism and social isolation among diverse older adults. It also helped form the Strategic Advisory Council, a group complementary to the CAB composed of community leaders, faculty, scholars, and policymakers, and charged with developing recommendations to address systemic inequities impacting the aging experience of diverse older adults. In 2024, the group entered its next phase, focused on strengthening the academic-community collaboration and aligning research priorities with those of the communities CHAI serves. CHAI will present key findings and highlights from each stage of its community-engagement process and will share lessons learned on its way to achieving its mission - the one of a truly community-engaged academic Center on Aging.

